# Betel quid use and mortality in Bangladesh: a cohort study

**DOI:** 10.2471/BLT.14.149484

**Published:** 2015-06-25

**Authors:** Fen Wu, Faruque Parvez, Tariqul Islam, Alauddin Ahmed, Muhammad Rakibuz-Zaman, Rabiul Hasan, Maria Argos, Diane Levy, Golam Sarwar, Habibul Ahsan, Yu Chen

**Affiliations:** a Department of Population Health, New York University School of Medicine, 650 First Avenue, New York, NY 10016, United States of America (USA).; b Department of Environmental Health Sciences, Columbia University, New York, USA.; c U-Chicago Research Bangladesh Ltd., Dhaka, Bangladesh.; d Department of Health Studies, University of Chicago, Chicago, USA.

## Abstract

**Objective:**

To evaluate the potential effects of betel quid chewing on mortality. (A quid consists of betel nut, wrapped in betel leaves; tobacco is added to the quid by some users).

**Methods:**

Prospective data were available on 20 033 individuals aged 18–75 years, living in Araihazar, Bangladesh. Demographic and exposure data were collected at baseline using a standardized questionnaire. Cause of death was defined by verbal autopsy questionnaires administered to next of kin. We estimated hazard ratios (HR) and their 95% confidence intervals (CI) for associations between betel use and mortality from all causes and from specific causes, using Cox proportional hazards models. We adjusted for age, sex, body mass index, educational attainment and tobacco smoking history.

**Findings:**

There were 1072 deaths during an average of 10 years of follow-up. Participants who had ever used betel were significantly more likely to die from all causes (HR: 1.26; 95% CI: 1.09–1.44) and cancer (HR: 1.55; 95% CI: 1.09–2.22); but not cardiovascular disease (HR: 1.16; 95% CI: 0.93–1.43). These findings were robust to adjustment for potential confounders. There was a dose–response relationship between mortality from all causes and both the duration and the intensity of betel use. The population attributable fraction for betel use was 14.1% for deaths from all causes and 24.2% for cancer.

**Conclusion:**

Betel quid use was associated with mortality from all causes and from cancer in this cohort.

## Résumé

**Objectif:**

Évaluer les effets potentiels de la consommation de chiques de bétel sur la mortalité. (Une chique se compose d'une noix de bétel enveloppée dans des feuilles de bétel; certains consommateurs lui ajoutent du tabac.)

**Méthodes:**

Des données prospectives étaient disponibles au sujet de 20 033 personnes âgées de 18 à 75 ans et vivant à Araihazar, au Bangladesh. Des données démographiques ainsi que des données sur l'exposition ont été recueillies au début de l'étude au moyen d'un questionnaire standardisé. Les causes de décès ont été définies à l'aide de questionnaires d'autopsie verbale auxquels ont répondu les proches. Nous avons estimé les ratios de risque (RR) et leur intervalle de confiance (IC) de 95% pour les associations entre la consommation de bétel et la mortalité, toutes causes confondues et due à des causes spécifiques, au moyen de modèles à risques proportionnels de Cox. Nous les avons ajustés en fonction de l'âge, du sexe, de l'indice de masse corporelle, du niveau d'instruction et des antécédents de tabagisme.

**Résultats:**

On a enregistré 1072 décès pendant une moyenne de 10 années de suivi. Les participants qui avaient consommé du bétel étaient sensiblement plus nombreux à mourir, de toutes causes (RR: 1,26; IC 95%: 1,09–1,44) et des suites d'un cancer (RR: 1,55; IC 95%: 1,09–2,22), mais pas de maladies cardiovasculaires (RR: 1,16; IC 95%: 0,93-1,43). Ces résultats sont restés identiques après ajustement en fonction d'éventuels facteurs de confusion. Une relation dose-effet a été observée entre la mortalité, toutes causes confondues, et la durée de la consommation de bétel ainsi que son intensité. La fraction attribuable dans la population concernant la consommation de bétel était de 14,1% pour les décès toutes causes confondues et de 24,2% pour les cancers.

**Conclusion:**

Dans cette cohorte, la consommation de chiques de bétel a été associée à la mortalité, toutes causes confondues et due au cancer.

## Resumen

**Objetivo:**

Evaluar los posibles efectos de mascar mascada de betel en la mortalidad. (Una mascada consiste de una nuez de betel envuelta en hojas de betel; algunos usuarios le añaden tabaco).

**Métodos:**

Se disponía de datos prospectivos de 20.033 individuos de entre 18 y 75 años que habitaban en Araihazar, Bangladesh. Se recogieron datos demográficos y de exposiciones al inicio del estudio mediante un cuestionaros normalizado. Se definió la causa de mortalidad a través de cuestionarios de autopsia verbales administrados a los familiares. Se estimó el cociente de riesgos y su intervalo de confianza (IC) del 95% para asociaciones entre el uso de betel y la mortalidad por todas las causas, y las causas específicas mediante el uso de modelos de Cox de riesgos proporcionales. Se ajustó en cuanto a edad, sexo, peso medio, nivel educativo e historial de consumo de tabaco.

**Resultados:**

Hubo 1.072 muertes en un periodo medio de seguimiento de 10 años. Los participantes que nunca habían utilizado betel tenían más posibilidades de morir por todas las causas (cociente de riesgos: 1,26; IC del 95%: 1,09–1,44) y cáncer (cociente de riesgos: 1,55; IC del 95%: 1,09–2,22); pero no enfermedades cardiovasculares (cociente de riesgos: 1,16; IC del 95%: 0,93–1,43). Estos resultados fueron sólidos para un ajuste de los factores potenciales de confusión. Hubo una relación de respuesta a la dosis entre la mortalidad de todas las causas y la duración e intensidad del uso de betel. La fracción atribuible de la población por el uso de betel fue del 14,1% de las muertes por todas las causas y del 24,2% por cáncer.

**Conclusión:**

El uso de mascada de betel estaba asociado a la mortalidad por todas las causas y por cáncer en este cohorte.

## ملخص

**الغرض:**

تقييم التأثيرات المحتملة لمضغ نبات التنبول على نسبة الوفيات. (تحتوي المُضغة على بذرة التنبول، الملفوفة في أوراق التنبول؛ وتتم إضافة مادة التبغ إلى المُضغة من جانب بعض المتعاطين).

**الطريقة:**

توفرت المعطيات الاستباقية عن 20033 شخصًا تتراوح أعمارهم ما بين 18 إلى 75 سنة ويعيشون في مدينة أرايهازار في بنغلاديش.

ولقد تم جمع معطيات ديموغرافية ومعطيات التعرض عند خط الأساس باستخدام استبيان معياري.

تم تحديد سبب الوفاة عن طريق استبيانات تشريح الجثة السردي التي تم تقديمها إلى الأقارب. وقد قمنا بتقدير نسب الخطورة (HR) ونسب أرجحيتها (CI) بمقدار 95% لعلاقات الاقتران بين تعاطي التنبول ونسب الوفيات الناتجة عن جميع الأسباب، أو الناتجة عن أسباب معينة، وذلك باستخدام نماذج المخاطر التناسبية لكوكس (Cox proportional hazards models). ولقد قمنا بالتعديل بحسب العمر، والجنس، ومؤشر كتلة الجسم، والتحصيل التعليمي، والتاريخ السابق لتدخين التبغ.

**النتائج:**

وقعت 1072 حالة وفاة خلال فترة متابعة تبلغ في المتوسط 10 سنوات. وكان المشاركون الذين تعاطوا التنبول من قبل أكثر عرضة للوفاة من جميع الأسباب بشكل ملحوظ (نسبة الخطورة: 1.26؛ بنسبة أرجحية مقدارها 95%: 1.09–1.44) وجراء الإصابة بمرض السرطان (نسبة الخطورة: 1.55؛ بنسبة أرجحية مقدارها 95%: 1.09–2.22)؛ ولكن ليس جراء الإصابة بمرض القلب الوعائي (نسبة الخطورة: 1.16؛ بنسبة أرجحية مقدارها 95%: 0.93–1.43). كانت هذه النتائج صامدة أمام التعديل للعوامل المحيّرة (Confounders) المحتملة. لقد كانت هناك علاقة بين الجرعة والاستجابة بين الوفيات الناتجة عن جميع الأسباب وكلاً من المدة الزمنية ودرجة شدة تعاطي التنبول. وبلغت النسبة المنسوبة إلى الشريحة السكانية من متعاطي التنبول 14.1% لحالات الوفاة الناتجة عن جميع الأسباب و24% من مرض السرطان.

**الاستنتاج:**

لقد كان تعاطي مُضغة التنبول مرتبطًا بالوفيات الناتجة عن جميع الأسباب وعن مرض السرطان في هذه المجموعة.

## 摘要

**目的:**

旨在评估嚼食槟榔对死亡率的潜在影响。 （槟榔嚼块由槟榔子组成，包裹在槟榔叶中，某些食用者会在槟榔嚼块中添加烟叶。）

**方法:**

分析居住在孟加拉国 Araihazar 18 岁到 75 岁的 20 033 个人的前瞻性数据。 使用标准化的调查问卷收集基线人口和接触数据。 通过向近亲进行死因推断问卷调查来确定死亡原因。 我们使用考克斯比例风险模型，评估食用槟榔与各种原因和特殊原因导致死亡率之间的危害比 (HR)，并且置信区间 (CI) 为 95%。 我们根据年龄、性别、身体质量指数、受教育程度和吸烟史调整群组。

**结果:**

在平均十年的随访病例中，有 1072 例死亡。 食用过槟榔嚼块的参与者明显更有可能死于各种原因 (HR: 1.26; 95% CI: 1.09–1.44) 和癌症 (HR: 1.55; 95% CI: 1.09–2.22)；而非心血管疾病 (HR: 1.16; 95% CI: 0.93–1.43)。这些结果强有力地支持潜在混杂因素的调整。 在各种原因导致的死亡率和槟郎食用的持续时间与强度之间存在剂量反应关系。 食用槟榔的人群归因分值中，各种原因导致的死亡占 14.1%，癌症导致的死亡占 24.2%。

**结论:**

在这个群组中，食用槟榔和各种原因以及癌症导致的死亡率有关。

## Резюме

**Цель:**

Оценить потенциальное воздействие жевания бетеля на смертность. Бетелевая жвачка состоит из ядра семени пальмы арека, завернутого в листья бетеля. Также к нему может добавляться табак.

**Методы:**

Были изучены проспективные данные 20 033 лиц в возрасте от 18 до 75 лет, проживающих в г. Араихазар, Бангладеш. Базовые демографические данные и сведения об употреблении бетеля были получены из стандартизированной анкеты. Причина смерти определялась с помощью анкетирования, основанного на вербальной аутопсии, вопросы из которого предъявлялись ближайшим родственникам покойного. Нами были оценены относительные риски (ОР) и их доверительные интервалы (ДИ) в 95%. Это позволило выявить взаимосвязь между жеванием бетеля и смертностью по любым причинам и по конкретным причинам. Для оценки использовалась пропорциональная модель рисков Кокса. Результаты были откорректированы с учетом возраста, пола, индекса массы тела (ИМТ), уровня образования и наличия табакокурения в анамнезе.

**Результаты:**

На протяжении 10 лет наблюдения умерли 1072 участника исследования. Участники, жевавшие бетель или хотя бы пробовавшие его, имели значительно более высокую вероятность умереть по какой-либо причине (ОР: 1,26; 95% ДИ: 1,09–1,44), в том числе от рака (ОР: 1,55; 95% ДИ: 1,09–2,22), но не от сердечно-сосудистых заболеваний (ОР: 1,16; 95% ДИ: 0,93–1,43). Эти выводы оказались устойчивыми к корректировке по всем потенциальным искажающим факторам. Между смертностью по какой-либо причине и длительностью, а также интенсивностью употребления бетеля наблюдалась зависимость «доза-эффект». Доля представителей населения, употребляющих бетель, составила 14,1% для смертей по всех остальным причинам и 24,2% для смертей от рака.

**Вывод:**

В данном когортном исследовании употребление бетелевой жвачки ассоциируется со смертностью от рака и по всем остальным причинам.

## Introduction

*Areca catechu* nut (betel nut) is the fourth most commonly used addictive substance in the world, after caffeine, nicotine and alcohol.^1^ Betel nut is consumed by chewing, either alone or in the form of a quid wrapped in betel leaves, slaked lime (calcium hydroxide) and different flavourings. Tobacco is added to the quid by some users. It is estimated that 600 million people worldwide chew betel regularly.^1^^,^^2^ Betel is widely used throughout central, south and south-east Asia, as well as in some South Pacific islands. With the growing number of immigrants from those areas, betel use is increasing in Africa, Australia, Europe and north America,^1^ where betel use remains an under-recognized public health issue.

The International Agency for Research on Cancer has concluded that betel without tobacco causes oral cancer, while betel with tobacco causes upper aerodigestive tract cancers, including cancer of the oral cavity, pharynx and oesophagus.^2^ Betel use has also been linked to metabolic syndrome,^3^^,^^4^ hypertension,^5^^,^^6^ diabetes mellitus,^7^^,^^8^ and obesity^9^^,^^10^ – diseases that are closely related to the development of cardiovascular disease (CVD). Recent evidence also suggests that betel use may play a role in CVD.^11^^–^^13^ Given that betel use has been related to an array of health outcomes, it is important to assess its impact on mortality.

Betel use has been a popular traditional habit in Bangladesh. According to a 2009 survey targeting all men and women aged 15 years or more in Bangladesh, betel was used by both men (23.5%) and women (25.2%).^14^ A study of the health effects of arsenic has recruited over 20 000 participants since the year 2000.^15^ This population-based cohort has also been used to assess other health issues.^16^^,^^17^ Here, we examine the association of betel use with mortality from all causes and from specific causes in this cohort.

## Methods

### Study population

A population-based survey was used to enumerate the sampling frame and characterize residents of a 25 km^2^ area in Araihazar, Bangladesh. Between October 2000 and May 2002, we recruited 11 746 participants who met the following eligibility criteria: married (to reduce loss to follow-up); aged 18–75 years; user of a tube well as a primary water supply and living in the study area for at least five years before recruitment.^15^ During 2006–2008, the cohort was expanded to include an additional 8287 participants (the expansion cohort) in the same study area following the same methods.^18^^–^^21^ The overall participation rate was 97%.

The cohort has been followed up with in-person home visits at 2-year intervals.^22^ Participants who were not at home during the first visit were revisited and excluded if they were not reachable during any of the three attempted visits. A field clinic was established exclusively for the participants and their family members to passively follow-up the participants between their biennial visits.^15^ Since this rural population lacks basic health-care services from the existing health-care facilities, all participants and their family members come to the clinic for all health-care needs. Informed consent was obtained from the study participants and study procedures were approved by the Ethical Committee of the Bangladesh Medical Research Council and the Institutional Review Boards of Columbia University and the University of Chicago.

### Questionnaire data

Social and demographic data were collected at baseline using a standardized questionnaire. Physicians measured height, weight and blood pressure with standard equipment.^23^^–^^25^ We asked participants if they had been diagnosed with diabetes and compared their answers with results from glycosylated haemoglobin and glucosuria tests.^26^ Questions on tobacco smoking included cigarettes and bidis (filterless, locally-produced cigarettes), smoked alone or together, past and current use and duration of tobacco smoking. To estimate the intensity of tobacco smoking, we calculated pack-years (the product of cigarettes or bidis smoked per day and years of smoking, divided by 20). Details of betel use were collected for both the original and expansion cohort, including information on past and current use, the number of times per day betel was used and years of betel use. Information on whether betel was chewed with smokeless tobacco was collected in the expansion cohort only. We did not collect information on the amount of smokeless tobacco that was used with betel. We also estimated the intensity of betel use (quid-years) as the product of times used per day and years of use.

### Assessment of mortality

The vital status of the participants was assessed at each follow-up home visit. Details of the assessment of causes of death are described elsewhere.^22^^,^^23^^,^^27^ Briefly, we adapted a validated verbal autopsy procedure that was developed by the International Centre for Diarrhoea Disease Research, Bangladesh (ICDDR, B), in collaboration with the World Health Organization (WHO). The method has been used to ascertain causes of death since 1971^28^ and has documented an overall 95% specificity, with an 85% sensitivity for deaths from cancer or CVD.^29^ During follow-up, upon receipt of a death reported by family or neighbours, a study physician and a trained social worker administered the verbal autopsy questionnaire to the next of kin. Medical records and death certificates were collected and reviewed monthly by an outcome-assessment committee, consisting of physicians and consulting medical specialists. Causes of death were coded according to the International Classification of Diseases, 10th Revision (ICD-10).^30^

### Statistical analyses

We computed person-years of follow-up from baseline to the date of death (for those who died) or to 21 April 2014. We estimated hazard ratios (HR) and 95% confidence intervals (CIs) for deaths from all causes, cancers and CVD using Cox proportional hazards models. History of betel use was classified as follows: never versus ever users; daily frequency (never, ≤ 3 times, 3–5 times and > 5 times); duration (never, ≤ 4 years, 4–12 years and > 12 years) and intensity (never, ≤ 12 quid-years, 13–60 quid-years and > 60 quid-years).We also estimated HRs for coronary heart disease, stroke and for cancer of the digestive or respiratory organs (these are the largest two categories of cancer deaths in the cohort).

We excluded 34 participants with missing data on betel use. Missing data on any of the covariates (8–281) were coded with dummy variables, allowing participants with missing data to be included in the analyses under a missing-at-random assumption. We first adjusted for sex and baseline age (model 1), we then adjusted for baseline body mass index (BMI; kg/m^2^), educational attainment (years) and smoking status (never, past and current), (model 2). The final model (model 3) was the same as model 2, except that we used intensity of tobacco smoking (pack-years) as the variable controlling for effects of tobacco.

Sensitivity analyses were conducted separately for mortality from all causes, cancers and CVD. We tested exclusion of deaths that occurred within two years of the baseline, under the assumption that individuals who are seriously ill at baseline are more likely to die in the first two years of follow-up. We conducted stratified analyses by sex, age, BMI, smoking status (never/ever) and educational attainment, adjusted for the same covariates as in model 2. In these models, we included interaction terms between betel (never versus ever user) and the dichotomous strata variables.

In the expansion cohort (*n* = 8287), we assessed whether mortality from all causes differed depending on whether chewing tobacco was included in the betel quids used. Finally, we tested inclusion of additional variables for arsenic exposure, systolic blood pressure and diabetes status in the models. We calculated the population attributable fraction (PAF) of mortality from all causes and from cancer associated with use of betel (ever users versus never users) using the following equation^17^:
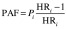
where *P_i_* is the proportion of mortality within the exposure category *i* and HR*_i_* is the adjusted HR of the *i*th category relative to the unexposed category. All analyses were done using SAS, version 9.3 (SAS Institute Inc., Cary, United States of America).

## Results

### Baseline characteristics

We observed 202 874 person-years during an average of 10 years of follow-up. There were 1072 deaths, of which 167 were from cancers and 439 were from CVD, together accounting for 56.5% of deaths. Among the deaths from CVD, 181 were from coronary heart disease and 183 were from stroke. Detailed causes of death and ICD-10 codes are shown in Table 1. Diabetes status as ascertained by questionnaire appeared valid, based on comparison with glycosylated haemoglobin and glucosuria tests. The prevalence of diabetes at baseline in this lean population was under 2%.^26^

**Table 1:** Underlying causes of death in the prospective study on betel use and mortality, Bangladesh, 2000–2014

Cause of death (ICD–10 code)	No.
**All causes**	1072
**Infectious and parasitic diseases (A00–B99)**	66
Tuberculosis (A15–A19)	32
Other bacterial diseases (A35, A40, A41)	9
Viral hepatitis (B16, B18, B19)	5
Sequelae of infectious and parasitic diseases (B90)	13
Other (A08, A09, A82, A91, B01)	7
**Cancer (C00–C97)**	167
Lip, oral cavity and pharynx (C02, C03, C09, C10, C13)	7
Digestive organs (C15–C26)	64
Stomach (C16)	17
Liver (C22)	32
Gallbladder (C23)	8
Other (C15, C18-C21)	7
Respiratory and intrathoracic organs (C30–C39)	53
Lung (C34)	46
Larynx (C32)	7
Female genital organs (C53, C55, C56)	9
Urinary tract (C64, C66, C67)	11
Ill-defined, secondary and unspecified sites (C76–C79)	5
Lymphoid, haematopoietic and related tissue (C85, C91, C92)	7
Other (C43, C49, C50, C61, C69, C71, C73)	11
**Diabetes mellitus (E10, E11, E14)**	10
**Diseases of the nervous system (G00–G99)**	12
Inflammatory diseases of the central nervous system (G00, G02, G04, G06)	6
Other (G20, G41, G45, G61, G91, G95)	6
**Cardiovascular diseases (I00–I99)**	439
Chronic rheumatic heart diseases (I05, I06, I08)	12
Ischaemic heart diseases (I21, I24, I25)	181
Other forms of heart disease (I35, I42, I46, I47, I50)	51
Stroke (I60-I64, I69)	183
Other (I11, I27, I73)	12
**Diseases of the respiratory system (J00–J99)**	144
Other chronic obstructive pulmonary disease (J44)	106
Asthma (J45)	17
Status asthmaticus (J46)	10
Other (J22, J41, J69, J90, J95)	11
**Diseases of the digestive system (K00–K93)**	54
Oesophagus, stomach and duodenum (K22, K25, K27, K29, K31)	7
Liver (K70–K72, K74, K76)	39
Other (K56, K63, K65, K80, K92)	8
**Diseases of the genitourinary system (N00–N99)**	26
Renal failure (N17, N18)	21
Other (N05, N13, N83, N93)	5
**Pregnancy, childbirth and the puerperium (O00–O99)**	15
Eclampsia (O15)	5
Complications of labour and delivery (O64, O71, O72, O75)	8
Other (O07, O95)	2
**Symptoms, signs and abnormal clinical and laboratory findings, not elsewhere classified (R00–R99)**	72
General symptoms and signs (R50, R54, R57)	6
Ill-defined and unknown causes of mortality (R96, R99)	62
Other (R10, R14, R90)	4
**External causes of morbidity and mortality (V01–Y98)**	41
Pedestrian injured in transport accident (V02–V04)	10
Intentional self-harm (X68, X70)	7
Assault (X90, X91, Y05, Y09)	5
Other (V33, V34, V80, V89, W14, W30, W70, W74, W86, W87, Y21, Y83)	19
**All other causes (D32, D37, D38, D43, D61, D64, D75, E64, E87, F20, L89, M51, M80, S06, T61, T82)**	26

The prevalence of past and current use of betel was 2.3% (465/19 999) and 32.7% (6535/19 999), respectively, in the overall study population. Given that the number of past users of betel was small, past and current users were combined as ever users in the analyses. While past and current use was significantly more frequent among men, women reported more frequent and intense use than men. Distributions of baseline variables by status of betel use are shown in Table 2. Past users were more likely to be men whereas more women were current users. Both past and current users tended to be older, less educated, past or current tobacco smokers and were more likely to have high BMI, high blood pressure or diabetes.

**Table 2:** Characteristics of participants, Bangladesh, 2000–2014

Characteristic	No. (%)		
	Betel use		
	Never (*n* = 12 999)	Past (*n* = 465)	Current (*n* = 6 535)
**Sex**			
Men	5 010 (38.5)	288 (61.9)	2 850 (43.6)
Women	7 989 (61.5)	177 (38.1)	3 685 (56.4)
**Age, years**			
18–29	5 199 (40.0)	25 (5.4)	426 (6.5)
30–39	4 590 (35.3)	106 (22.8)	1 754 (26.8)
40–49	2 260 (17.4)	138 (29.6)	2 529 (38.7)
≥ 50	950 (7.3)	196 (42.2)	1 826 (27.9)
**BMI, kg/m^2^**			
< 18.5	4 832 (37.6)	199 (43.5)	2 750 (42.8)
18.5–24.9	7 047 (54.9)	224 (49.0)	3 266 (50.8)
> 24.9	958 (7.5)	34 (7.4)	410 (6.4)
**Education, years**			
None	4 837 (37.2)	230 (49.5)	3 647 (55.8)
1–5	4 114 (31.7)	136 (29.2)	1 849 (28.3)
6–9	2 346 (18.1)	59 (12.7)	611 (9.4)
≥ 10	1 694 (13.0)	40 (8.6)	425 (6.5)
**Cigarette/bidi use**			
Never	9 793 (75.4)	179 (38.5)	3 519 (53.9)
Past	273 (2.1)	75 (16.1)	906 (13.9)
Current	2 929 (22.5)	211 (45.4)	2 109 (32.3)
**SBP, mm/Hg**			
< 140	11 938 (92.9)	388 (84.5)	5 727 (89.0)
≥ 140	907 (7.1)	71 (15.5)	711 (11.0)
**DBP**, **mm/Hg**			
< 90	11 707 (91.2)	402 (87.8)	5 787 (89.9)
≥ 90	1 133 (8.8)	56 (12.2)	649 (10.1)
**Diabetes**			
Yes	178 (1.4)	21 (4.7)	144 (2.2)
No	12 619 (98.6)	428 (95.3)	6 290 (97.8)

BMI: body mass index; DBP: diastolic blood pressure; SBP: systolic blood pressure.

Note: Data were missing on betel use for 34 subjects; on BMI for 281 subjects; on education for 11 subjects; on cigarette/bidi smoking for 8 subjects; on systolic blood pressure for 260 subjects; on diastolic blood pressure for 268 subjects; and on diabetes status for 321 subjects.

### Betel use and mortality

Betel use was positively associated with all-cause and cancer-related mortality, after adjusting for age and sex (model 1). The associations did not change substantially when potential confounders (BMI, smoking status and educational attainment) were added to the model (model 2) or after adjusting for intensity of tobacco smoking (model 3, Table 3). Associations were significant for all causes of death (HR: 1.26; 95% CI: 1.09–1.44) and for cancer (HR: 1.55; 95% CI: 1.09–2.22); but not for CVD (HR: 1.16; 95% CI: 0.93–1.43).

**Table 3:** Betel use and mortality, Bangladesh, 2000–2014

Variable	Person-years	All causes			Cancers			CVD	
		No.	HR (95% CI)		No.	HR (95% CI)		No.	HR (95% CI)
**Betel use**									
Never	130 808	422	1.00		62	1.00		174	1.00
Ever	71 669	646	1.26 (1.09–1.44)		105	1.55 (1.09–2.22)		265	1.16 (0.93–1.43)
Excluding deaths in 1–2 years of follow-up	71 572	560	1.30 (1.12–1.51)		89	1.59 (1.08–2.33)		236	1.18 (0.94–1.48)
**Frequency of use**									
Never	130 808	422	1.00		62	1.00		174	1.00
≤ 3 times/day	29 990	292	1.38 (1.17–1.63)		48	1.67 (1.10–2.53)		129	1.40 (1.09–1.80)
3–5 times/day	19 645	156	1.17 (0.96–1.43)		27	1.53 (0.94–2.50)		63	1.06 (0.77–1.45)
> 5 times/day	21 787	196	1.16 (0.96–1.40)		30	1.42 (0.89–2.29)		72	0.92 (0.68–1.25)
**Duration of use**									
Never	130 808	422	1.00		62	1.00		174	1.00
≤ 4 years	28 130	177	1.16 (0.96–1.40)		35	1.73 (1.11–2.70)		67	1.05 (0.77–1.43)
4–12 years	22 311	186	1.24 (1.03–1.49)		32	1.59 (1.00–2.52)		75	1.16 (0.87–1.55)
> 12 years	20 708	278	1.37 (1.15–1.62)		38	1.38 (0.87–2.19)		120	1.22 (0.93–1.58)
**Intensity of use**									
Never	130 808	422	1.00		62	1.00			1.00
≤ 12 quid-years	24 617	158	1.24 (1.02–1.51)		28	1.64 (1.02–2.64)		62	1.21 (0.88–1.65)
13–60 quid-years	26 077	237	1.19 (0.99–1.41)		44	1.51 (1.05–2.49)		97	1.10 (0.83–1.44)
> 60 quid-years	20 442	246	1.35 (1.13–1.62)		33	1.44 (0.90–2.29)		103	1.17 (0.89–1.54)

CI: confidence interval; CVD: cardiovascular disease; HR: hazard ratio.

Note: Cox proportional hazards models, adjusting for sex, baseline age, educational attainment, body mass index and pack-years of tobacco smoking.

The results were not appreciably altered by inclusion of arsenic exposure, systolic blood pressure or diabetes status in the models, or by exclusion of deaths reported in the first 2 years of follow-up (Table 3). The population attributable fraction for betel use was 14.1% for deaths from all causes and 24.2% for cancer-related deaths.

We observed a dose–response relationship between mortality from all causes and duration of betel use. Among participants who had used betel for less than 4 years, for 4–12 years and for more than 12 years, the HRs were 1.16 (95% CI: 0.96–1.40), 1.24 (95% CI: 1.03–1.49) and 1.37 (95% CI: 1.15–1.62) respectively (Table 3). Results for intensity of use were similar, though the central estimates of risk did not increase monotonically with intensity of use. A dose–response relationship was not evident for mortality from cancers or CVD (Table 3).

Betel use was associated with mortality from cancer of the digestive organs after adjustment for age and sex (HR: 1.92; 95% CI: 1.09–3.36). However, this association was attenuated and no longer significant after further adjusting for BMI, smoking status and educational attainment (HR: 1.70; 95% CI: 0.96–2.99). The risk of death from respiratory cancers was also increased among ever users, but not significantly (HR: 1.75; 95% CI: 0.96–3.18). There were no significant associations between betel use and the risk of death from coronary heart disease or stroke (data available from the corresponding author).

### Subgroup analyses

The association between betel use and mortality from all causes was stronger in younger individuals as well as in individuals with a higher BMI; however, these interactions did not reach statistical significance (*P* for interaction = 0.06 and 0.09, respectively). Associations between betel use and mortality from all causes and from cancer did not differ substantially by sex or by smoking status. For instance, the HR for mortality from all causes was 1.24 (95% CI: 0.97–1.58) among never smokers and 1.19 (95% CI: 1.01–1.40) among ever smokers. Similarly, the HR for cancer mortality was 1.47 (95% CI: 0.78–2.76) among never smokers and 1.38 (95% CI: 0.92–2.07) among ever smokers.

In the expansion cohort, we had information on whether or not betel was chewed with tobacco. Among 2541 ever users, 2042 (80.4%) chewed betel with tobacco, while 499 (19.6%) chewed betel without tobacco. The association between betel use and mortality from all causes persisted and was marginally significant among individuals who chewed betel without tobacco (HR: 1.55; 95% CI: 0.99–2.44). Surprisingly, there was no significant association between betel use and mortality among those who chewed betel with tobacco (HR: 0.93; 95% CI: 0.65–1.32). There was a similar proportion of tobacco smokers among people who used betel alone and those who also used chewing tobacco (40.4% and 41.3%, respectively).

**Fig. 1:**
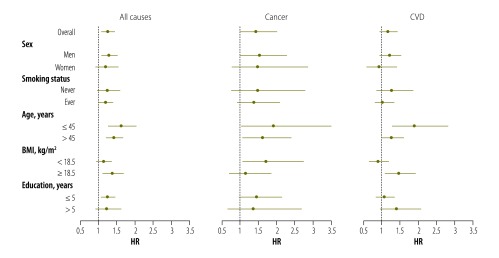
Betel use and mortality, Bangladesh, 2000–2014

BMI: body mass index, CVD: cardiovascular disease; HR: hazard ratio.

Note: Cox proportional hazards models were adjusted for baseline age, sex, body mass index, smoking status (never, past and current) and educational attainment. For the stratified models, we included an interaction term between betel use and the dichotomous strata variable.

For mortality from CVD, there was a significant interaction between betel use and age (*P* = 0.01) and between betel use and BMI (*P* = 0.02), such that the risk was higher among younger individuals (HR: 1.88; 95% CI: 1.27–2.80) and those with a higher BMI (HR: 1.44; 95% CI: 1.08–1.91) relative to older individuals or those with a lower BMI (Fig. 1).

## Discussion

Betel use was significantly associated with all-cause and cancer-related mortality in this south Asian cohort. Two studies in India reported mixed results for chewing of betel quid or betel nut (without tobacco added).^31^^,^^32^ One study^31^ reported no significant effect of chewing betel quid, while the other reported increased all-cause mortality with age-adjusted relative risks of 1.19 among women and 1.11 among men,^32^ but confidence intervals were not reported. In China, a study reported increased all-cause mortality associated with betel nut chewing and smoking.^33^ Betel use was associated with all-cause mortality in two other studies, with relative risks of 1.19 and 1.40, respectively.^12^^,^^13^ Mortality increased with higher frequency of betel use,^13^ longer duration of use^12^ or greater intensity of use (quid-years).^12^ Increased risk was predominantly seen in users who had chewed betel for 25 years or for 350 quid-years or longer.^12^

We report increased mortality with longer duration and intensity of use, but not with higher frequency of use. In our cohort, the median duration and intensity of betel use were seven years and 30 quid-years, respectively. It is possible that the dose–response relationship is not apparent at lower intensity of use, or that this cohort has too limited a range of exposure to detect a dose–response relationship. In China, it was reported that betel was mostly used by men and rarely by women and almost all users were smokers,^33^ in contrast to our study population in which men and women had a similar prevalence of betel use and less than half of betel users were also smokers.

Several previous studies investigated the effects of betel use on the risk of death from all cancers or cancer of the oral cavity and other upper-digestive organs. There was increased mortality from cancer of the oral cavity, nasopharynx, liver and lung associated with betel nut chewing and smoking in China.^33^ In another study, betel use without chewing tobacco was associated with a significant increase in deaths from all cancers and cancer of the oesophagus, liver, pancreas, larynx and lung but despite the fact that 90% of the betel users were also smokers, the authors did not control for smoking status.^34^ Another cohort study in an elderly population found no association between betel use and cancer-related mortality.^12^

In our study, we controlled for smoking and found a significantly higher risk of cancer-related mortality in participants who had ever used betel. The effect estimates did not differ substantially by smoking status. Only seven deaths in the cohort were reportedly due to cancer of the oral cavity and other upper digestive organs and analyses excluding these cases did not change the effect estimates. The associations with cancer were similar in men and women, although not significant in women. Taken together, our data add to the growing body of evidence of the influence of betel use on cancer and suggest that the effect may be independent of tobacco smoking.

Several prospective studies have suggested an overall positive association between betel use and CVD risk,^11^^–^^13^ although some other studies found no association when CVD subtypes were considered.^12^^,^^33^ In a prospective cohort of 6511 men older than 50 years, users of betel nut were at a higher risk of mortality from overall CVD and stroke but not coronary heart disease.^12^ In a cohort of 56 116 men, betel nut chewing was independently associated with a greater risk of CVD mortality.^13^ Similarly, in a prospective registry-based cohort study of 21 906 men followed for 2.7 years, an independent dose–response effect of betel use on risk of incident CVD cases and deaths was observed.^11^ Although we found no significant association between betel use and CVD mortality overall, CVD mortality was significantly increased among younger people and those with a higher BMI. In our previous analyses of betel use and blood pressure in the same cohort, we found that betel use without tobacco was associated with higher blood pressure.^5^

Betel contains four arecal alkaloids (primarily arecoline, along with arecaidine, guvacine and guvacoline), all of which have been shown to produce nitrosamine derivatives that have potential carcinogenic effects.^2^ Betel components can induce both local and systemic release of inflammatory biomarkers^35^^–^^37^ thereby provoking oxidative stress^38^ and chronic inflammation related to the development of systemic diseases. Betel chewing also causes periodontal disease,^39^^,^^40^ a risk factor for cancer and CVD.^41^^,^^42^

Our study represents a large population from south Asia that has received little epidemiologic attention. Other strengths of the present study include the population-based prospective study design with a high response rate (97%) and the extensive data on betel use and potential confounders, including smoking status and the intensity of smoking.

Several potential limitations, however, should also be noted. First, this cohort was not established to focus on betel use and thus is lacking information on changes in use over time. Also the population was relatively young, with a mean age of 36 years at baseline and the overall average duration of use was relatively short. The relatively small number of cases for subtypes of cancer or CVD may explain the insignificant effect estimates on these outcomes.

Second, we did not have comprehensive data on chewing tobacco use, either alone or with betel. Research on the effects of chewing tobacco (in the absence of betel nut) on mortality has been inconclusive.^43^^,^^44^ In one study, no association was observed between chewing betel either with or without tobacco and mortality.^31^ In our cohort, the positive association for all-cause mortality remained among individuals who did not use smokeless tobacco together with betel, but no significant association was observed among those who chewed betel with tobacco. However, a limited number of subjects had data on whether chewing tobacco was used together with betel and larger studies are needed. Lastly, we cannot rule out the possibility of residual confounding by tobacco smoking in our findings.

Our data suggest that betel has a small-to-moderate impact on mortality from all causes and from cancer in this Bangladeshi population. Future larger studies are warranted to investigate the effects of betel use on subtypes of cancer and CVD.
